# Platelet-rich plasma for the treatment of postoperative anastomotic fistula of the gastrointestinal tract: a case report

**DOI:** 10.3389/fphar.2026.1727021

**Published:** 2026-02-11

**Authors:** Sihui Jia, Yawen Guo, Changkai Zhang, Xiaoyuan Sun, Licun Wang, Haiyan Wang

**Affiliations:** Department of Blood Transfusion, The Affiliated Hospital of Qingdao University, Qingdao, China

**Keywords:** case report, intestinal leakage, platelet-rich plasma (PRP), postoperative anastomotic fistula, wound healing

## Abstract

**Introduction:**

Anastomotic fistulas are a possible complication of local digestive tract resections, and their main symptoms include abdominal pain, peritonitis, and infections. Currently, the treatment of anastomotic fistulas is primarily conservative or involves surgical repair. Platelet-rich plasma (PRP) contains high concentrations of platelets and is used to promote wound healing. However, the use of PRP in the treatment of anastomotic fistulas has rarely been reported.

**Case presentation:**

The patient was a 63 year-old man, Chinese and Asian. The patient underwent radical treatment for a malignant colon tumor in 2011. In 2014, he complained of persistent abdominal pain and was diagnosed with a malignant gastric tumor for which he underwent a subtotal (Billroth I) gastrectomy. After the surgery, an anastomotic fistula was observed. After more than 1 month of conservative treatment, the patient’s anastomotic fistula still failed to heal; then, autologous PRP was attempted. PRP was prepared by centrifugation and activated into a gel state. The fistula was treated with PRP gel occlusion endoscopically, and a total of 3 mL was injected. One week after treatment, the fistula was healed.

**Diagnosis:**

Anastomotic fistula after subtotal gastrectomy.

**Interventions:**

Platelet-rich plasma (PRP) gel occluded anastomotic fistula under gastroscopy.

**Outcomes:**

One week after PRP treatment, the closure of the fistula was observed.

**Conclusion:**

PRP derived from the patient’s own blood, offers advantages for healing intestinal fistulas that fail to respond to prolonged conservative therapy. Injecting PRP gel directly into these fistulas promotes tissue regeneration and closure.

## 1 Introduction

An anastomotic fistula refers to the leakage or rupture of two cavity organs (such as the intestine, esophagus, or stomach) after suture or anastomosis during surgery, resulting in the leakage of contents (such as digestive fluid, food, or feces) into the surrounding tissues or body cavity. This is a serious postoperative complication that may cause infections, abscesses, and peritonitis, and may even be life-threatening. The incidence of anastomotic fistulas after esophageal and pancreatic surgery is relatively high, reaching 5%–20%, whereas the incidence of anastomotic fistulas after gastric, small intestinal, and colorectal surgeries is less than 5%. Patients with postoperative symptoms of unexplained abdominal pain, peritonitis (tenderness, rebound tenderness, and muscle tension) (Shao et al., 2020), or systemic symptoms (Søreide et al., 2015) suggestive of infections should undergo imaging examinations and serum inflammatory marker tests to identify possible anastomotic fistulas. Postoperative anastomotic fistulas account for 75%–85% of all fistulas in the gastrointestinal tract caused by unintentional enterotomies, anastomotic site rupture, proximal anastomotic foreign bodies, poor anastomotic techniques, distal obstructions, hematomas, anastomotic site abscess formation, or tumors. Conservative treatment, endoscopic treatment, and surgical treatment can be selected for the management of anastomotic fistulas (Wang et al., 2024). For minor internal fistulas or intestinal perforations, conservative treatment (Nur et al., 2023) is mainly used, including fasting, gastrointestinal decompression, antimicrobial therapy (Nur et al., 2023), providing a healing environment, and waiting for the wound to heal (Søreide et al., 2015). Passive closure using endoscopic biological forceps can also be performed for appropriate intestinal perforation lesions (Schmidt et al., 2016; Farah et al., 2024). When the scope of the fistula or perforation is large, or when the above two treatment methods are not suitable, surgery for secondary closure of the fistula should be performed (Nascimbeni et al., 2021).

PRP, a type of autologous platelet plasma concentration created by centrifugation, contains high levels of growth factors (Pinto et al., 2014; Paichitrojjana and Paichitrojjana, 2022) and cytokines (Paichitrojjana and Paichitrojjana, 2022; Suthar et al., 2017) that play key roles in inflammation and tissue repair (Everts et al., 2006) by promoting hemostasis at the site of vascular injury. There are many types of growth factors, such as fibroblast growth factor (FGF), transforming growth factor β (TGF-β) (Zhang et al., 2025), platelet-derived growth factor (PDGF) (Zhang et al., 2025; Andersen et al., 2021), epidermal growth factor (EGF) (Andersen et al., 2021), vascular endothelial growth factor (VEGF) (Zhang et al., 2025; Qiu et al., 2024), platelet growth factor (PGF, platelet-derived) (Pinto et al., 2014; Paichitrojjana and Paichitrojjana, 2022; He et al., 2023; Azam et al., 2024; Everts et al., 2024; Sharara et al., 2021), among others (Liang et al., 2023a). Platelets initiate the wound healing process by releasing locally active growth factors (Siddique et al., 2022) that can stimulate matrix metalloproteinase synthesis, promote fibroblast proliferation (Siddique et al., 2022), enhance extracellular matrix production (He et al., 2023), and accelerate tissue healing and regeneration (Liang et al., 2023b). These growth factors attract undifferentiated cells to the injury site and promote their cell division (Azam et al., 2024). PRP significantly enhances the healing process (Siddique et al., 2022), is used to treat a variety of conditions, and has been successful in several medical fields, including wound healing (Suthar et al., 2017; Qiu et al., 2024; He et al., 2023; Azam et al., 2024; Everts et al., 2024; Pineda-Cortel et al., 2023), bone regeneration (Everts et al., 2006; He et al., 2023; Pineda-Cortel et al., 2023), osteoarthritis (He et al., 2023; Liang et al., 2023b; Pineda-Cortel et al., 2023), hair follicle regeneration (Paichitrojjana and Paichitrojjana, 2022; Qiu et al., 2024; Pineda-Cortel et al., 2023), body tissue repair (Everts et al., 2006; He et al., 2023; Everts et al., 2024; Pineda-Cortel et al., 2023; Liang et al., 2022), and skin regeneration (Qiu et al., 2024; Pineda-Cortel et al., 2023).

To date, there have been no reports on the use of PRP to treat anastomotic fistulas after gastrointestinal surgery. The following is a case of the introduction of PRP gel in the treatment of an anastomotic fistula.

## 2 Case presentation

### 2.1 Patient

The patient was a 63 year-old man who underwent radical resection of a malignant tumor of the ascending colon in 2014. Seven years after the surgery, the patient developed symptoms of acid reflux and heartburn, which lasted for 2 months, without obvious causes. The patient underwent complete gastroenteroscopy at the Yantai Laiyang Central Hospital on January 8, 2021, which revealed elevated lesions in the gastric fundus and anterior wall of the gastric antrum. The histopathological results indicated that the small curvature of the anterior wall of the gastric antrum showed differentiated adenocarcinoma, chronic mucosal inflammation in the anterior wall of the proximal gastric antrum, and low-grade intraepithelial neoplasia in the epithelium of the mucosal glands in the posterior wall of the gastric fundus. The patient was admitted to the Emergency General Surgery Department of our hospital for surgical treatment on January 17, 2021. A routine physical examination revealed no obvious abnormalities upon admission. However, one concern was that the patient had a history of diabetes mellitus for more than 10 years.

After admission, the patient was diagnosed with gastric adenocarcinoma based on his clinical manifestations, gastroenteroscopy findings, and histopathological findings. After an improved preoperative examination, the patient underwent a subtotal gastrectomy (Billroth I) under general anesthesia. Unfortunately, the patient developed pain in the upper abdomen, chest, waist, and back on postoperative day three. Imaging revealed no significant abnormalities, and the patient was discharged from the hospital on January 27, 2021.

**FIGURE 1:**
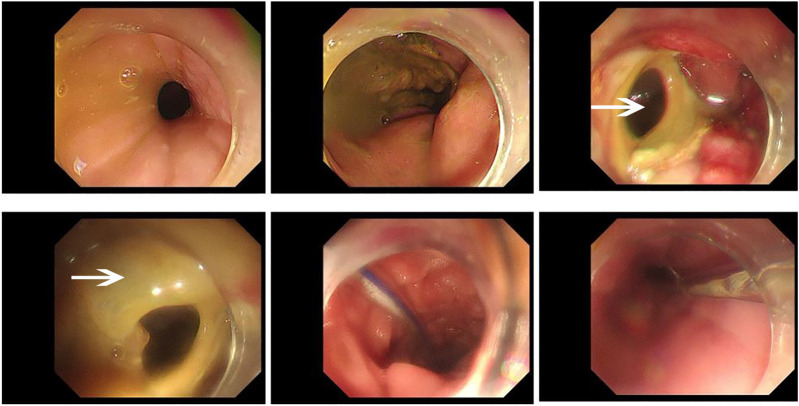
Gastroscopy revealed the presence of a postoperative anastomotic fistula on February 9, 2021. During the gastroscopy procedure, a moderate amount of yellow, viscous material could be observed, and the enteral nutrition tube could also be seen. As shown by the arrow in the figure, the fistula can be seen in the anterior wall of the stomach, and the surrounding mucosa is hyperemia and edema.

After discharge, the patient developed pain and tenderness over the entire abdomen, nausea, and vomiting (which consisted of food content). The patient was admitted to the Emergency General Surgery department of our hospital on February 2, 2021. After admission, abdominal computed tomography (CT) revealed a patchy area of mixed density around the gastric anastomosis, with changes in gas and fluid levels. Combined with the patient’s clinical manifestations, an anastomotic fistula was diagnosed. Conservative treatments, such as antimicrobial therapy, fasting, intravenous nutrition, and abdominal drainage, were implemented immediately to improve the patient’s general condition. After one week of conservative treatment, the patient underwent angiography after oral administration of a contrast medium, and leakage at the anastomotic site was observed on February 8 (Figure 4A). The patient underwent a gastroscopy with local anesthesia, and a nasogastric tube was inserted on February 9. In addition, hyperemia and edema of the surrounding mucosa were observed on gastroscopy (Figure 1). The conservative treatment was continued. The patient received enteral nutritional support, and abdominal drainage was performed simultaneously. On February 18, upper gastrointestinal radiography reexamination showed that the fistula was smaller than before but was still not closed (Figure 4B). After conservative treatment for another week, the fistula did not close; therefore, PRP gel plugging under endoscopy was performed.

### 2.2 PRP treatment

#### 2.2.1 PRP preparation

PRP preparation, 200 mL of whole blood was extracted into a sterile triple blood bag containing 3.8% sodium citrate and was centrifuged at 22 °C ± 2 °C at 1500r/min for 12 min. The blood in the bag was divided into three layers (Liang et al., 2022) after centrifugation: platelet-poor plasma (PPP) and PRP (Suthar et al., 2017) were separated from red blood cells (Paichitrojjana and Paichitrojjana, 2022; Everts et al., 2006). PPP and PRP were separated (Liang et al., 2023b) into the second bag via a serous separator, and then centrifuged at 22 °C ± 2 °C for 3000r/min for 15 min to separate the PRP and PPP. The PRP final product was suspended in about 15 mL of plasma. Next, it was incubated at 22 °C ± 2 °C for 1–2 h to get the platelets back in suspension (Liang et al., 2023a; Liu et al., 2022). On February 26, the baseline platelet concentration of the patient was 173 x 10^9^/L. The platelet count of the PRP end-product was 658 × 10^9^/L, which was 3.8 times the baseline platelet concentration (Siddique et al., 2022; Smith and Rai, 2024). 500 U of freeze-dried thrombin powder (He et al., 2023) and 1 mL of 10% calcium chloride was used to prepare the activators under sterile conditions (Everts et al., 2006). The PRP gel was prepared *in vitro* by activating PRP with medical bovine thrombin (Paichitrojjana and Paichitrojjana, 2022; Everts et al., 2006) (PRP-to-activator ratio, 10:1) (Andersen et al., 2021; Sharara et al., 2021; Liang et al., 2023b).

#### 2.2.2 PRP gel fistula closure

**FIGURE 2:**
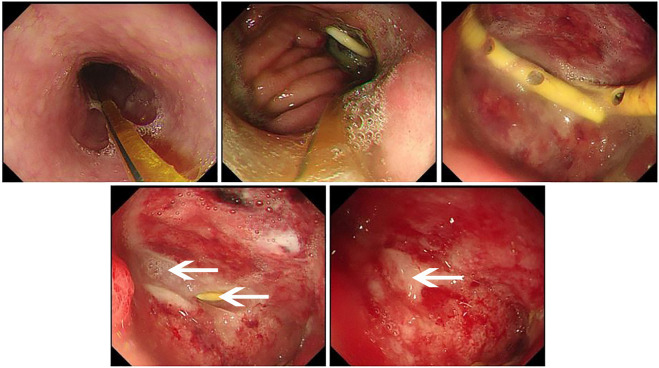
The PRP sealing treatment was performed under gastroscopy observation on February 26, 2021. Under the gastroscopy, the enteral nutrition tube and the yellow pigtail tube used for PRP occlusion treatment could be observed. As shown by the arrow in the figure, 3 mL PRP gel was injected into the orifice of fistula to seal the orifice. The drainage tube and white gel were visible in the figure.

**FIGURE 3:**
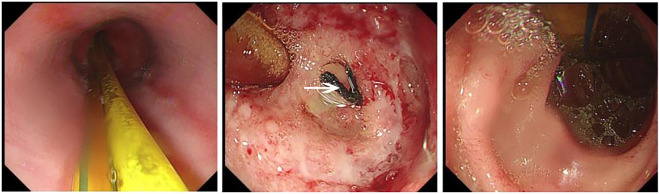
The fistula healed after PRP treatment on March 5, 2021. Under the gastroscopy, the enteral nutrition tube can be seen. As shown by the arrow in the figure, scar after fistula healing was found on the anterior wall of the anastomosis one week after surgery, and the surrounding tissue was hyperemia and edema. Good filling was observed after CO_2_ injection into the residual stomach.

**FIGURE 4:**
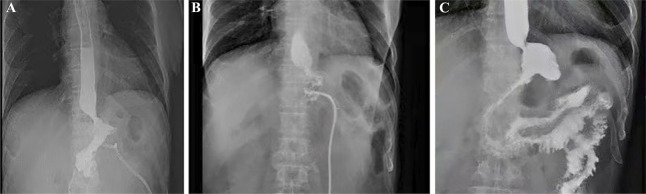
Results of upper gastrointestinal contrast examination. **(A)** On February 5, 2021, the patient underwent upper gastrointestinal radiography, and the contrast agent could be seen leaking from the fistula. **(B)** On February 18, 2021, the patient underwent upper gastrointestinal radiography, and the contrast agent could be seen entering the esophagus from the fistula. **(C)** On March 10, 2021, the patient underwent upper gastrointestinal radiography again, and no leakage of contrast agent was observed.

PRP gel fistula closure and gastroscopic tube insertion were performed On February 26, 2021. During the procedure, a total of 3 mL of autologous PRP gel was injected through an abdominal drainage tube (Figure 2). Postoperative fasting, acid suppression, gastrointestinal decompression, and other supportive treatments were continued after the gastroscopy treatment. Approximately one week after PRP treatment, repeat gastroscopy was performed, and a postoperative fistula healing scar was found on the anterior wall of the gastric anastomosis (Figure 3). The anastomosis was still hyperemic and edematous, but the residual stomach was well-filled after CO_2_ insufflation (Figure 3). At the same time, the upper abdominal CT examination showed that the anastomotic wall was thickened and the gas shadow was visible; however, it was better than before. Two weeks after the PRP treatment, a contrast meal and follow-through were performed. The fistula was completely closed, and the anastomosis was unobstructed (Figure 4C).

#### 2.2.3 Thoughts on treatment methods

The patient is a 63 year-old male with a history of diabetes for over 10 years and has previously undergone two major surgeries (classified as Grade III or above). According to his blood glucose records, glycemic control has been unstable. Following gastric resection, he developed symptoms of anastomotic leakage within the first postoperative week. During a subsequent hospitalization, he also presented with fever and vomiting. Despite receiving both parenteral and enteral nutrition during fasting periods, his blood glucose levels remained poorly controlled. Given the patient’s overall condition, surgical intervention was deemed unsuitable. Furthermore, both the patient and his family expressed a preference for a safer, less invasive treatment approach. At the time, daily abdominal drainage output was 20–30 mL. We therefore proposed injecting PRP through the drainage tube into the fistula tract to promote healing. After discussion with the patient, he expressed willingness to try this approach.

## 3 Discussion and conclusion

The patient in this case had a history of colon cancer treated more than 10 years before this presentation. After surgery for colon cancer, intestinal function weakens, and the blood supply changes. The patient was diagnosed with gastric cancer 7 years after the surgery for the colon cancer, and chose to undergo surgical intervention again. The potential causes of the postoperative anastomotic fistula in this patient include iatrogenic injury related to surgical manipulation and problems with intestinal function and blood supply after colon cancer surgery. In addition, the patient had chronic diabetes mellitus, and long-term high blood glucose levels are also an important factor causing wound healing problems (Suthar et al., 2017; Smith and Rai, 2024). The postoperative anastomotic fistula is believed to have occurred because of the above-mentioned factors, and conservative treatment was unsuccessful.

We believe that when conventional treatment methods are ineffective, PRP has the potential to be applied in the repair treatment of complex intestinal fistulas, which can help reduce the difficulty of treatment, increase the healing rate, and minimize complications. Its application has the following advantages: on the one hand, PRP is derived from the patient’s own body (Zhang et al., 2025), which can reduce the risk of immune rejection (Liang et al., 2022) and infections (Suthar et al., 2017); moreover, it avoids the occurrence of adverse reactions caused by drug application or surgical procedures, making it highly safe and with few adverse effects (Paichitrojjana and Paichitrojjana, 2022; Zhang et al., 2025; Pineda-Cortel et al., 2023; Liang et al., 2022). PRP contains white blood cells and antibacterial proteins, such as platelet factor 4 (PF-4) (He et al., 2023), connective tissue activating peptide III (CTAP-III) (He et al., 2023), and pre-platelet basic protein (PPBP). Due to its antibacterial (Nascimbeni et al., 2021; Suthar et al., 2017; Azam et al., 2024; Smith and Rai, 2024) effect, it is expected to reduce the use of antibiotics and lower the probability of abdominal cavity infection (He et al., 2023). At the same time, PRP is rich in various growth factors, which itself has the functions of promoting repair and anti-inflammatory (Paichitrojjana and Paichitrojjana, 2022; Zhang et al., 2025; Liang et al., 2023b; Liang et al., 2022), and can increase the first-stage healing rate and reduce the recurrence rate. On the other hand, PRP is easy to obtain (Suthar et al., 2017; Zhang et al., 2025), has the characteristics of convenient administration, small trauma, and high safety, which can reduce the probability of secondary surgery, and most of patient can have PRP prepared with autologous blood. If extreme cases occur, allogeneic PRP can be used to complete the treatment (Liang et al., 2022). Previous studies have proved that the use of allogeneic PRP is feasible. In 2007, European researchers first described the application of allogeneic PRP in a case of delayed fracture healing. (Smrke et al., 2007). Subsequently, in 2010, Italian scientists separately reported favorable outcomes using allogeneic PRP in two clinical settings: 115 cases of finger trauma and 17 cases of skin ulcers of varying etiologies (Balbo et al., 2010; Bernuzzi et al., 2010).

Platelet-rich plasma is emerging as a promising conservative therapy for fistula, particularly in elderly patients, individuals with chronic diseases, and those unsuitable for surgical intervention. PRP is rich in platelets, which release a variety of bioactive factors upon activation. These factors promote tissue healing, modulate inflammatory responses, exhibit antibacterial properties, and demonstrate good biocompatibility, all of which provide a theoretical rationale for PRP as an ideal material for fistula closure. Given its clinical advantages, PRP is anticipated to become a leading treatment option for refractory wounds. This approach significantly shortens the treatment duration of gastrointestinal fistulas, reduces hospital length of stay and dependence on parenteral nutrition, and consequently decreases the incidence of complications related to prolonged immobility. For patients, it also lowers treatment costs and alleviates both physical and psychological burdens. The fibrin matrix formed by the gel carrier serves as a protective barrier against bacterial contamination while additionally supporting tissue proliferation during wound healing.

However, clinical studies on PRP for gastrointestinal fistula remain limited. Currently, fistula occlusion is primarily considered for low-output external fistulas, and standardized guidelines for optimal PRP concentration, dosage, and treatment frequency are still lacking. Importantly, PRP therapy must be administered alongside foundational medical care, including adequate drainage of exudate, correction of electrolyte imbalances, and comprehensive nutritional support.

Currently, PRP is widely used because it contains a variety of growth factors (Pinto et al., 2014) and has been applied in fracture repair (Andersen et al., 2021), reproductive organ recovery (Paichitrojjana and Paichitrojjana, 2022; Ivaskiene et al., 2025; Lin et al., 2021), joint cavity repair (Pineda-Cortel et al., 2023; Liang et al., 2022), and surgical wound repair (Suthar et al., 2017). Within the scope of internal fistulas, it has also been reported that PRP can be used to repair bronchial fistulas. One case report has described the treatment of a bronchial fistula after lung transplantation using PRP gel under bronchoscopy. In the reported case, 50 mL of PRP was applied twice, and bronchoscopy performed on the 50th postoperative day showed that the anastomotic fistula was completely closed and healed. There were no bronchial fistulas or other complications at later follow-ups (Siddique et al., 2022). PRP treatment for skin ulcers has also been studied (Suthar et al., 2017; Qiu et al., 2024; Liu et al., 2022). In the treatment of skin ulcers, repeated treatment can show that the wound begins to heal in approximately 4 weeks, and complete healing takes approximately 8 weeks (Suthar et al., 2017). Additional studies have investigated the promotion of bone tissue healing by PRP (Everts et al., 2006; Andersen et al., 2021; Pineda-Cortel et al., 2023), demonstrating that morphogenetic growth factors involved in bone growth can transform undifferentiated mesenchymal stem cells (MSCs), a type of adult multipotent stem cell, into immature and mature bone progenitor cells through bone morphogenetic proteins (BMPs) (Everts et al., 2006; Andersen et al., 2021). These BMP growth factors belong to the TGF-β superfamily and are also present in PRP. Most PGFs in PRP exert mitogenic effects that increase the number of healing cells through mitosis (Everts et al., 2006). PRP plays an increasingly important role in vivo and *in vitro* repair therapies owing to its various advantages.

After the patient was discharged, we conducted a follow-up call. The patient did not exhibit any symptoms consistent with an anastomotic fistula, and did not return to our hospital for further imaging examinations. Although the effect of PRP treatment in this case was significant, it is still difficult to use PRP as a routine treatment. Further precision and standardization are required in terms of the mode of administration, dose, and frequency of treatment (Zhang et al., 2025). In this case, the amount of PRP injected into the intestinal fistula was mainly based on the size and depth of the fistula; the nutritional status of the patient was not taken into account. For example, the hyperglycemic state environment in the body of patients with diabetes mellitus affects the process of wound healing, and at present, the dosage of PRP lacks a standardized therapeutic dosage, which needs to be further clarified to determine whether single or multiple doses are required (Andersen et al., 2021). In the treatment of open wounds, it is also necessary to consider the nature of the patient’s skin, whether it easily forms scars or whether wound healing is slow, and personalized dosages should be determined for different situations. When used in small amounts, it may lead to poor wound healing, prolonged healing times, and weak skin/tissue at the wound site. When the dosage is high, autologous blood collection prepared using PRP correspondingly increases the blood supply cost for the patient (Zhang et al., 2025). If the wound area is large, autologous blood is frequently collected for PRP preparation in a short period of time. The preparation of autologous PRP may be challenging in certain patients, such as those with hematological disorders, malignant tumors, or individuals on medications that reduce platelet survival. In these scenarios, the use of allogeneic PRP may be considered as a potential alternative (Liang et al., 2022; Barrionuevo et al., 2015). Furthermore, there are currently few clinically available reported cases of PRP usage for the treatment of intestinal fistulas, and individual differences may exist, resulting in a certain degree of chance in the treatment results. In the future, more laboratory data and clinical applications are needed to confirm the noninvasive healing effect of PRP.

## Data Availability

The original contributions presented in the study are included in the article/supplementary material, further inquiries can be directed to the corresponding author.
